# Cooperative regulation of myosin-S1 binding to actin filaments by a continuous flexible Tm–Tn chain

**DOI:** 10.1007/s00249-012-0859-8

**Published:** 2012-10-07

**Authors:** Srboljub M. Mijailovich, Oliver Kayser-Herold, Xiaochuan Li, Hugh Griffiths, Michael A. Geeves

**Affiliations:** 1 Department of Environmental Health, Harvard School of Public Health, Boston, MA 02115 USA; 2 Department of Biosciences, University of Kent, Canterbury, Kent CT2 7NJ UK; 3 Department of Medicine, School of Medicine, Tufts University, CBR, Room 406, Boston, MA 02135 USA; 4 Steward St. Elizabeth’s Medical Center, Boston, MA 02135 USA

**Keywords:** Tropomyosin chain, Myosin-S1 binding, Ca^2+^ sensitivity, Stopped flow

## Abstract

The regulation of striated muscle contraction involves cooperative interactions between actin filaments, myosin-S1 (S1), tropomyosin (Tm), troponin (Tn), and calcium. These interactions are modeled by treating overlapping tropomyosins as a continuous flexible chain (CFC), weakly confined by electrostatic interactions with actin. The CFC is displaced locally in opposite directions on the actin surface by the binding of either S1 or Troponin I (TnI) to actin. The apparent rate constants for myosin and TnI binding to and detachment from actin are then intrinsically coupled via the CFC model to the presence of neighboring bound S1s and TnIs. Monte Carlo simulations at prescribed values of the CFC stiffness, the CFC’s degree of azimuthal confinement, and the angular displacements caused by the bound proteins were able to predict the stopped-flow transients of S1 binding to regulated F-actin. The transients collected over a large range of calcium concentrations could be well described by adjusting a single calcium-dependent parameter, the rate constant of TnI detachment from actin, *k*_−I_. The resulting equilibrium constant $K_{\text{B}} \equiv 1/K_{\text{I}}$ varied sigmoidally with the free calcium, increasing from 0.12 at low calcium (pCa >7) to 12 at high calcium (pCa <5.5) with a Hill coefficient of ~2.15. The similarity of the curves for excess-actin and excess-myosin data confirms their allosteric relationship. The spatially explicit calculations confirmed variable sizes for the cooperative units and clustering of bound myosins at low calcium concentrations. Moreover, inclusion of negative cooperativity between myosin units predicted the observed slowing of myosin binding at excess-myosin concentrations.

**Electronic supplementary material:**

The online version of this article (doi:10.1007/s00249-012-0859-8) contains supplementary material, which is available to authorized users.

## Introduction

The contractility of striated muscle is regulated by calcium-dependent azimuthal movements of tropomyosin–troponin complexes over the surface of the actin filament. In the steric blocking model (Squire 1981), the affinity of myosin for regulated F-actin is controlled by tropomyosin (Tm), where each Tm protomer spans seven actin sites on the same strand of the actin double helix. This fact alone suggests an explanation for the observed autocooperativity of myosin binding (Bremel and Weber 1972; Greene and Eisenberg 1980; Lehrer and Morris 1982; Metzger 1995; Tobacman and Butters 2000), because the binding of one myosin displaces the tropomyosin to a new azimuthal orientation which allows myosin binding to the other six actin sites. While this two-state model is consistent with a variety of experiments (Gordon et al. 2000), the fact that myosin weakly binds to actin in the absence of calcium suggests that this model is incomplete and that a third state, in which weak myosin binding is permitted, might be necessary. Based on studies of myosin-S1 binding to regulated F-actin in solution, McKillop and Geeves proposed a model with three regulatory states: blocked, closed, and open (McKillop and Geeves 1993). The blocked state prohibits any myosin binding, the closed state permits weak myosin binding as observed at low Ca^2+^, and the open state permits both weak and strong myosin binding. The corresponding azimuthal orientations of tropomyosin were also observed in cryo-electron microscopy (cryo-EM) studies (Vibert et al. 1997), and they were designated by Lehman et al. (2000) as B (blocked), C (calcium-induced), and M (myosin-induced). In the McKillop–Geeves notation, C and M states correspond to the closed and open states, respectively.

An assumption common to these regulatory models (Gordon et al. 2000; McKillop and Geeves 1993) is that each tropomyosin molecule can be treated as a rigid unit moving between two or three discrete orientations, each generating different actin affinities for myosin. Thus, the size of the cooperative unit was structurally defined by the length of one tropomyosin molecule. However, affinity data for myosin binding in solution showed that the size of the functional cooperative unit could be very different from the 40 nm size of one tropomyosin molecule (Maytum et al. 1999). Moreover, structural data show that adjacent tropomyosins along the thin filament overlap slightly (Lorenz et al. 1995; Murakami et al. 2008; Palm et al. 2003; Vibert et al. 1997). Thus, the assumption of independently acting cooperative units is not viable. A model with attractive end-to-end interactions between tropomyosins was formulated by Hill et al. (1980), accounting for the possibility that all tropomyosins along one strand of F-actin form a chain of interacting rigid units with two possible orientations. In this model, the rigid unit moving between two discrete orientations generates different actin affinities for myosin and the weak end-to-end interactions between adjacent tropomyosins favor states (i.e., Tm positions on actin) of the same kind. Alternatively, the bending compliance of an individual Tm may be extended via the linked ends. In this case, the elastic bending compliance of the assembly is located in the overlap of Tm–Tm regions linked to the tail of Troponin T (TnT) so that the assembly can be viewed as a continuous flexible chain (CFC) (Lehrer et al. 1997). Convincing evidence for the latter model comes from recent cryo-EM studies with increased axial resolution (Pirani et al. 2005) which show some tropomyosins lying between the three orientations previously observed.

In the CFC model developed by Smith et al. (Smith 2001; Smith and Geeves 2003; Smith et al. 2003) the closed state of the McKillop–Geeves model (McKillop and Geeves 1993) is replaced by a global state of all thermally excited configurations of the bent chain. Each bound myosin after isomerization generates a locally defined open state where the chain is displaced away from its resting orientation and the resulting kink in the chain facilitates additional myosin binding to other sites under the kink. The size of the kink, analogous to the size of the cooperative unit, is at least twice the persistence length of the confined chain. This cooperativity is a consequence of axial propagation of the chain displacements along the actin filament strand by one or more strongly bound myosin-S1s.

**Fig. 1:**
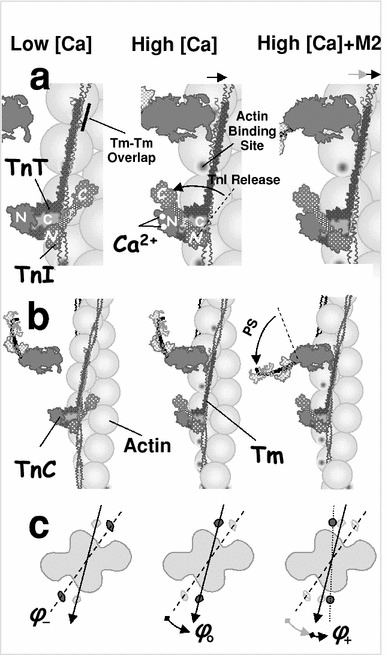
Schematic structure of the regulated A-Tm–Tn filament, showing a single chain of sequentially connected Tm molecules. TnT is bound to one end of Tm, and its N-terminus overlaps the adjacent Tm. The C-terminus of TnC and the N-terminal of TnI are bound to TnT. At low calcium, the C-terminal of TnI is bound to actin, fixing the Tm chain at an azimuthal angle *ϕ*_−_. At this position, the Tm chain overlaps the actin binding site and blocks myosin binding (**a**, *left*). When two calcium ions are bound to TnC (+Ca^2+^), the hands of the N-terminal region of TnC are open and bind a region of TnI near its C-terminus, reducing affinity of TnI to actin. The TnI rotates away from its actin binding site and allows that Tm units only weakly interact with actin. The Tm moves toward its equilibrium position at *ϕ*_o_, partially opening the actin site (**a**, *middle*), permitting weak myosin binding to actin (**b**, *middle*). Thermally induced fluctuations in the azimuthal angle of Tm permit strong myosin binding to actin, displacing the Tm chain to *ϕ*_+_. The arrows on the *top* of (**a**) denote the Tm shifts after binding Ca^2+^ and strong binding of myosin respectively (from *left* to *right*). The azimuthal movement of Tm on the actin surface and three characteristic angular positions are denoted in (**c**). Based on models of Gagne et al. (1995) and Tripet et al. (1997)

At low calcium, myosin binding is inhibited through the troponin attached to each Tm because the Tm chain is azimuthally displaced into a local blocked state by TnI bound to actin (Fig. 1). There may also be cooperative inhibition when the persistence length of the chain exceeds the 36 nm spacing between adjacent bound Tns. At high calcium, two calcium ions bind to a (skeletal muscle) Tn, and the Tn undergoes an allosteric transition, causing its detachment from actin (Gagne et al. 1995; Tripet et al. 1997). After release of Tn from actin, the unconstrained Tm chain moves into its energetically favorable position, i.e., into the closed state. The CFC model has been tested against myosin equilibrium binding data in solution (Smith and Geeves 2003; Smith et al. 2003), and it was found that calcium-regulated binding of TnI to actin provides a reasonably complete model of thin filament regulation in solution.

To further investigate the applicability of the CFC model to data in solution we developed a Monte Carlo method to simulate the time course of myosin binding to actin as observed in stopped-flow mixing experiments. To do this, the CFC model is first used to predict the regulation of myosin–actin binding rates as well as myosin–actin affinity. The basic idea is that thermal fluctuations in the CFC orientation are much faster than myosin kinetics, so the effective binding rate is regulated by the fraction of time in which the CFC has moved past the closed angle *ϕ*_o_ (Fig. 1). This time fraction is controlled by nearby chain orientations, and will be increased if myosins are bound to adjacent actin sites or decreased if nearby Tns are bound to actin. In our Monte Carlo procedure, the state of all actin monomers along F-actin strands (free, occupied by myosin or by troponin) are specified and tracked in time through a stochastic implementation of the underlying kinetics. Thus, the CFC model in conjunction with Monte Carlo methods provides a theoretical framework for evaluating thin filament regulation of actin–myosin interactions, including the various cooperativities: (1) myosin–Tm CFC cooperativity imposed by accelerated strong myosin binding, (2) myosin binding inhibition by Tn–Tn cooperativity, and (3) slowing down of myosin binding observed at later stages of stopped-flow transients when the myosin concentration exceeds that of actin.

## Methods

Following the approach of Smith et al. for modeling thin filament regulation as a CFC (Geeves et al. 2011; Smith 2001; Smith and Geeves 2003; Smith et al. 2003), we developed a Monte Carlo algorithm to quantify spatially explicit myosin-S1 binding to regulated F-actin in solution. The algorithm consists of three main steps: (1) calculation of the state transitions between actin–Tn states as a function of [Ca^2+^], (2) calculation of mean CFC angular positions and their azimuthal fluctuations along the actin filament, and (3) calculation of state transitions between actin-S1 states. These processes are interrelated and regulated by the calcium concentration. The CFC position and its fluctuations modulate the original state transition rates of Tn binding to actin or myosin-S1 binding to actin, whereas the spatial position of pinning sites at which the Tn and myosin-S1 are bound to actin determines the mean Tm chain angular position and the thermally induced azimuthal fluctuations.

Structurally, the Tm molecule is a coiled coil of about 40 nm in length, covers seven monomers on one strand of the actin double helix, and is associated with one Tn molecule. On each strand of F-actin, the TmTn units overlap and interact with the adjacent units, forming the appearance of a CFC (Lorenz et al. 1993; Vibert et al. 1997). The associated troponin molecule consists of three subcomponents: troponin T, troponin C and troponin I, denoted as TnT, TnC, and TnI, respectively (Fig. 1). One end of TnT is bound to a specific site on Tm, and its N-terminus overlaps the adjacent Tm. In the absence of calcium the N-terminal region of TnC is closed and the C-terminal of adjacent TnI is bound to actin, prohibiting Tm movement, i.e., holding Tm in the “blocked” state. Thus, in relaxed muscle, TnI holds Tm in an azimuthal position *ϕ*_−_ that sterically blocks myosin-S1 binding sites on F-actin. In the presence of Ca^2+^, binding of one or two Ca^2+^ ions to TnC generates a conformational change in TnI, lowering the affinity of TnI to F-actin. The release of the TnI C-terminus from F-actin now allows the unconstrained Tm chain to move toward the “closed” state, i.e., the azimuthal position *ϕ*_o_, favoring myosin binding to F-actin (Fig. 1) and, therefore, muscle contraction (Smith and Geeves 2003; Vibert et al. 1997).

The flexibility of the Tm chain and its weak interaction with F-actin permit thermally induced fluctuations in the azimuthal position of the Tm chain, except at the constrained (pinning) sites where TnI is bound to actin or when the CFC is displaced by bound myosin. The mean CFC angular position and its azimuthal fluctuations along the actin filament are, therefore, determined by the position of the pinning sites induced by TnI binding to actin and by myosin-S1 binding to actin. The azimuthal movement of the CFC chain around the current mean position of the Tm chain permits myosin binding to actin only at those instances in time when the actin sites are not blocked by the CFC. Therefore, the probability of this state transition at a specified spatial location along the actin filament is proportionally reduced. Thus, the dynamic movement of the continuous Tm chain on the actin surface modifies myosin binding rates in proportion to the fraction of time when the actin sites are available for binding. In turn, bound myosin heads and TnI bound to actin restrict the motion of the Tm chain and, therefore, affect the mean configuration of CFC and its fluctuations. Regulation by this scheme is a dynamic process that couples the reaction rates to the current CFC configuration, and this configuration explicitly depends on the spatial positions of currently bound myosin-S1s and TnIs. Even so, this system can be described as a Markov process^1^ if the time increments are sufficiently small that not more than one binding or unbinding transition per actin filament occurs within the current time step.

**Fig. 2:**
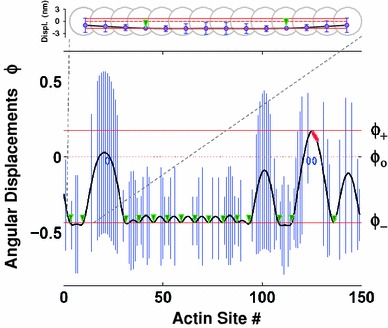
Mean and standard deviation (SD) of the angular displacements of CFC at calcium concentration of 6.6 μM at 0.17 s after injection of 0.25 μM myosin-S1 in 2.5 μM solution of F-actin. Mean angular displacements, $\bar{\phi }$, are depicted as *black line* and the SD, *σ*_*ϕ*_, is shown by *bars* (*blue*) at each actin site. The CFC is pinned at locations of bound TnIs at angle *ϕ*_−_ (*green triangles*), and of strongly bound myosin at angle *ϕ*_+_ (*red ovals*). At these locations, angular fluctuations are prohibited. *Red solid lines* denote bound troponin pinning angle *ϕ*_−_, and the position of the chain displaced by strongly bound myosin at *ϕ*_+_. *Red dashed line* denotes the position of the minimum of the confined energy well at *ϕ*_o_. The plotted angular displacements and their standard deviations are magnified compared with the distance between neighboring actin sites and exaggerate bending of the chain. The insert shows the displacements at the first 14 actin sites at the same scale as the size of the actin monomers, and thus the realistic bent shape of the CFC. The axial distance between the actin monomers is taken to be 5.5 nm. Model parameters used are presented in Table 1

For the simulation of myosin binding transients to regulated actin we set up a Monte Carlo algorithm considering each of the continuous tropomyosin chains on a single strand of actin monomers on F-actin as an independent subsystem. In this setting, tropomyosin is associated with the actin filament via a Tn molecule at every seventh monomer, starting from the third actin monomer from left to right (Fig. 2). Each actin filament contains two CFCs, and each CFC has the prescribed number of Tm–Tn complexes. The binding of TnI to actin fixes the continuous tropomyosin chain at the so-called TnI pinning points at an angle *ϕ*_−_. The number of TnI pinning points depends on the calcium concentration and is modulated by the number and position of bound myosin-S1s. Weak binding of myosin-S1 to actin blocks the CFC movement in an azimuthal direction toward *ϕ* ≤ *ϕ*_o_ and therefore reduces the rebinding rate of TnI to actin. At a fixed Ca^2+^ concentration, the spatial distribution of the positions of bound TnIs is a dynamic process and changes depending on the degree of Ca^2+^ binding to TnC and the subsequent release of TnI from actin. Another restriction on the CFC movement is imposed by the strongly bound myosin-S1s at azimuthal positions *ϕ*_+_ (Fig. 1).

### CFC angular position and its variance

We modeled Tm interactions with the actin surface in the presence of Tn via a loosely confined quasicontinuous semiflexible chain. The CFC spans one strand along the whole length of the actin filament (Fig. 1). For simplicity, the Tm chain is assumed to be elastically homogeneous, and the potential well that provides angular confinement has a single minimum in the absence of constraints imposed by myosin-S1 or Tn bound to actin. These assumptions provide the basis for the mathematical formulation and a qualitative description of how Tm regulates the energetics of myosin binding in the presence of Tn and varying calcium concentrations.

The displacements of the Tm–Tn chain are determined by the energy of chain kinks, the chain elasticity, and the energy landscape of the interaction of the Tm–Tn chain with actin. We use Feynman’s integral to calculate the potential energy of the Tm-Tn chain interacting with actin and myosin (Feynman and Hibbs 1965; Smith 2001). The governing equation of the continuous tropomyosin chain is the expression for the energy of a distorted chain of length *L* with angular displacement $\phi (s)$ at position *s*:

1 $$ E\left\{ {\phi (s)} \right\} = \int\limits_{0}^{L} {\left[ {\frac{\kappa }{2}\phi^{\prime \prime } (s)^{2} + \frac{\alpha }{2}\phi (s)^{2} } \right]{\text{d}}s}. $$

The parameter *κ* specifies a bending stiffness of the chain, and the parameter *α* specifies the curvature of a quadratic potential which serves to confine the chain to the “groove” between the strands of the actin double helix. The mean configuration of the closed state sits at the bottom of the confining potential at the angle *ϕ*_o_ = 0. At the positions where a myosin is strongly bound to actin, the chain is assumed to be pinned at an angle *ϕ*_+_ > 0, giving a local open state. Where a TnI on the chain is bound, the chain is pinned in the opposite direction at angle *ϕ*_−_ < 0, a local blocked state. The opposing signs guarantee that the blocked, closed, and open states correspond to the three orientations seen in cryo-EM micrographs.

The potential energy functional (Eq. 1) defines the path of minimum energy (Feynman and Hibbs 1965) for the confined worm-like chain with pinning sites of bound TnIs or myosin-S1s at defined axial positions along F-actin (Smith 2001). In between the pinning sites, the minimum energy path satisfies the corresponding Euler–Lagrange equation

2 $$ \left( {\frac{{{\text{d}}^{4} }}{{{\text{d}}s^{4} }} + 4\xi^{4} } \right)\phi (s) = 0 $$

where $\xi = (\alpha /4\kappa )^{1/4}$ is the inverse persistence length of the confined Tm–Tn chain. Because the energy expression is harmonic, the path of minimum energy is also the path of the mean CFC angle $\bar{\phi }(s)$ in an equilibrium distribution of thermally excited CFC configurations at absolute temperature *T*. The partition function for this distribution is a Feynman path integral or functional integral over the complete set of CFC configurations $\left\{ {\phi (s)} \right\}$, and can also be used to calculate the standard deviation *σ*(*s*) of CFC angles arising from thermal excitation. It is convenient to simplify these calculations by making a pair approximation, where the mean and standard deviation depend only on the distances to the nearest pinning sites on either side, at positions *s* − *x* and *s* + *y*. Here, *x* and *y* are the distances between the current position *s* along the CFC and the position of the left and the right pinning site, respectively. Each of these simple boundary pinning sites includes a pair of chain kinks depending on whether the kinks are produced by bound myosin or bound TnI. Analytic expressions for the functions $\bar{\phi }(x,y)$ and $\sigma_{\phi } (x,y)$ for known angular boundary displacements (*ϕ*_a_, *ϕ*_b_), slopes $(\phi_{\text{a}}^{\prime } ,\phi_{\text{b}}^{\prime })$ and the arc length of the chain segment, *ℓ*, are available (Smith 2001). The solution obtained for the nearest-neighbor boundary displacements can be for (*ϕ*_−_, *ϕ*_−_) or (*ϕ*_+_, *ϕ*_+_) for a symmetrical, or (*ϕ*_+_, *ϕ*_−_) for an asymmetrical configuration, where *ϕ*_−_ denotes the displacement imposed by bound TnI and *ϕ*_+_ denotes the displacement imposed by strongly bound myosin-S1. However, these expressions are too complex to display except in terms of intermediate functions. Computer subroutines for these functions and the pair interaction energies between kinks of symmetrical and asymmetrical configuration are given in the Electronic Supplementary Material reported in Geeves et al. (2011). The mean angle $\bar{\phi }(x,y)$ interpolates between the angles of each pinning site, which are recovered when *x* = 0 or *y* = 0. At a pinning site, the standard deviation *σ*_ϕ_(*x*, *y*) is zero, and as the sites are removed to distances beyond the persistence length, *σ*_ϕ_(*x*, *y*) approaches the standard deviation $\sigma_{\text{o}} = \left( {k_{\text{B}} T/8\kappa \xi^{3} } \right)^{{{1 \mathord{\left/ {\vphantom {1 2}} \right. \kern-0pt} 2}}}$ of the free chain (Smith et al. 2003).

The configuration of a whole CFC is defined by the positions of bound molecules of myosin-S1 and TnI and their respective angular displacements *ϕ*_+_ and *ϕ*_−_. Because the analytic expressions for the functions $\bar{\phi }(x,y)$ and *σ*_*ϕ*_(*x*, *y*) are derived for a single segment between two pinning sites, we divided the whole CFC length, *L*, into a sequence of *m* chain regions of length *ℓ*_*m*_, between any two nearest-neighbor pinning sites or a pinning site and free end of the CFC so that these analytic expressions could be used in calculations of the CFC configuration along the entire actin filament. Continuity of the CFC as a beamlike structure requires that *ϕ*(*s*) and its first and second derivatives, $\phi^{\prime } (s)$ and $\phi^{\prime \prime } (s)$, respectively, are smooth, differentiable functions along the whole CFC length spanning an actin filament. To satisfy these conditions, the piecewise solution can be constructed based on the functions $\bar{\phi }(x,y)$ and *σ*_*ϕ*_(*x*, *y*) if these functions satisfy compatibility conditions at common pinning points between two neighboring chain segments. The compatibility conditions require matching of the *ϕ*_*m*_(*s*), slope, and curvature of two neighboring segments. The piecewise semianalytic solution for $\bar{\phi }(x,y)$ along a single strand was verified numerically using a finite-element algorithm to solve a modified beam equation (Bathe 1996), which estimates the errors arising from neglecting three-site interactions, and is potentially more robust as it avoids the use of hyperbolic functions at large arguments. Multiple comparisons showed negligible differences between the solutions obtained by these two methods, confirming that either method can be used without compromising the accuracy of the solution of Eq. 2 for the prescribed boundary conditions.

### Chain-regulated kinetics

The current model features two biochemical Tn–actin states, one in which TnI is bound to actin and thus, in conjunction with TnT, maintains the position of Tm, inhibiting myosin-S1 binding, and the other where TnI is not bound to actin, thus allowing the Tm chain to move azimuthally along the actin surface. These biochemical states are associated with the three structural states, namely B (blocked), C (closed), and the open or M-state (Lehrer et al. 1997; Maytum et al. 1999; McKillop and Geeves 1993), and they are modulated by actin–myosin states which include free (unoccupied) actin sites, and weakly and strongly bound myosin-S1 states. The closed state is induced by the confined energy landscape, which mimics the electrostatic interaction between Tm and actin, and is located at the minimum of the confinement potential at *ϕ*_o_ = 0. When Tm is in the closed state, myosin-S1 can weakly bind to actin. This binding prevents movement of Tm for *ϕ* ≤ *ϕ*_o_ at that location, and the sparsely populated myosin-S1 state strictly prevents TnI rebinding if the TnI binding site is on the same actin monomer. Our model allows for two-step myosin binding to actin. The second step provides the transition from weak to strong binding, when *ϕ* ≥ *ϕ*_+_, forming the open state. This transition is rapid and rather irreversible.

We assume that only the rate constants of TnI binding to actin, *k*_I_, and of weak and strong S1 binding to actin, *k*_M1_ and *k*_M2_, respectively, are regulated by the CFC, while the detachment rates *k*_−M1_ and *k*_−M2_ are unregulated. The detachment rate of TnI from actin, *k*_−I_, is strictly regulated by Ca^2+^ and is not modulated by the CFC configuration. The regulated rate constants may be expressed in terms of multiplicative factors that represent the fraction of time that the CFC is at orientations that permit binding. This statement applies only if the CFC movements are rapid with respect to binding kinetics, as shown in Table 1. The response time of the CFC to local perturbations is certainly much less than the 1 ms resolution time of stopped-flow responses (Geeves and Lehrer 1994) and is expected to be of the order of 10^−5^ s. Thus, the CFC movements are very rapid compared with myosin binding rates (Geeves and Lehrer 1994), and probably also with respect to TnI binding.

**Table 1:** Values of the Tm–Tn chain parameters *κ*, *α*, *ξ*, and *σ*_o_ and the rates of state transition constants, estimated as described in the main text

Values of key model parameters		
Tropomyosin pinning angle	*ϕ*_−_	−25°
Myosin imposed Tm angular displacement	*ϕ*_+_	10°
Tropomyosin persistence length	$L_{{p{\text{Tm}}}}$	150 nm
Tropomyosin–troponin persistence length	$L_{{p{\text{TmTn}}}}$	250 nm
Bending stiffness of Tm–Tn chain per unit length	*κ*_TmTn_	9.87 × 10^2^ pN nm^2^
Angular Tm bending stiffness per unit length	*κ*	2.0 × 10^4^ pΝ nm^4^
Persistence length of Tm–Tn confined chain	1/*ξ*	22.2 nm
Angular standard deviation of free chain	*σ*_ο_	29.7°
Strength of the chain confining potential	*α*	0.341 pN
Myosin–actin binding rate	$k_{\text{M1}}^{\text{o}}$	4.2 × 10^6^ M^−1^ s^−1^
Myosin detachment rate	*k*_−M1_	20 s^−1^
Myosin stroke rate	$k_{\text{M2}}^{\text{o}}$	500 s^−1^
Reverse stroke rate	*k*_−M2_	5 s^−1^
TnI–actin binding rate	$k_{\text{I}}^{\text{o}}$	100 s^−1^
TnI–actin detachment rate at high Ca^2+^	*k*_−I_	~1,200 s^−1^

*κ* was obtained from the persistence length $L_{{p{\text{TmTn}}}}$ of the free TmTn complex in solution, using *R* = 4.5 nm for the radius at which tropomyosin sits on unregulated F-actin (Vibert et al. 1997; Xu et al. 1999). The persistence length 1/*ξ* applies to the confined chain on F-actin

The values for *ϕ*_−_ and *ϕ*_+_ are taken from (Poole et al. 2006; Vibert et al. 1997)

The estimate of *κ*_TmTn_ is obtained from the tropomyosin persistence length $L_{{p{\text{Tm}}}}$ in solution (Phillips and Chacko 1996; Sousa et al. 2010) and by scaling as (radius)^4^ from actin measurements (Yanagida et al. 1984; Yasuda et al. 1996) as reported earlier (Smith et al. 2003). Similar values for *κ*_TmTn_ are also reported in (Geeves et al. 2011), based on *L*_*p*Tm_ reported by (Li et al. 2010a; Li et al. 2010b)

The detachment of TnI from actin and the reattachment of TnI to actin are strongly dependent on the Ca^2+^ concentration. It is also likely that both the detachment and reattachment rates of TnI are strain dependent. For a specified Ca^2+^ concentration, the equilibrium constant, $K_{\text{I}}$, can, in principle, be obtained from the transient data. However, the strain-dependent kinetics of TnI binding to actin is complex due to (1) unknown and highly variable forces acting on TnI–actin bonds that can modulate TnI detachment or even cause the detachment without Ca^2+^ binding to TnC, and (2) due to the modulation of TnI reattachment probably caused by the nonlinear combination of CFC bending and interaction of TmTn units with actin.

Because the details of these processes are largely unknown, we implemented in our algorithm the following simplified approach: (1) the rate of TnI detachment, *k*_−I_ is a function of Ca^2+^ concentration only, and (2) the rate of TnI attachment to actin is a weighted function of the rate of TnI binding to actin from the closed state, $k_{\text{I}}^{\text{o}}$, i.e.,

3 $$ k_{\text{I}} (s_{i} ) = \frac{{r_{\text{I}} \left( {\bar{\phi }_{i} ,\sigma_{i} } \right)}}{{r_{\text{I}}^{\text{o}} \left( {\phi_{\text{o}} ,\sigma_{\text{o}} } \right)}}k_{\text{I}}^{\text{o}}. $$

Here, *s*_*i*_ denotes the discrete position of actin site *i* along an actin filament strand, and *σ*_*i*_ is the angular standard deviation of the CFC, *σ*_*ϕ*_ (*x*_*i*_, *y*_*i*_), at the location of site *i*. The attachment rate of TnI from the closed state is denoted as $k_{\text{I}}^{\text{o}}$. Assuming that the thermal motion follows a normal distribution around the CFC mean position (Smith 2001), the weighted functions are the probabilities that the CFC lies in the angular range *ϕ* ≤ *ϕ*_−_. These probabilities are represented as the cumulative forms of Gaussian distributions of the CFC angular positions at $\bar{\phi }_{i} \le \phi_{ - }$, with standard deviations *σ*_*i*_ and *σ*_o_, for both the current mean position of CFC, $\bar{\phi }_{i}$, and the closed position, *ϕ*_o_, respectively.

4 $$ \begin{aligned} r_{\text{I}} \left( {\bar{\phi }_{i} ,\sigma_{i} } \right) & = \tfrac{1}{2}\,{\text{erfc}}\left[ {{{\left( {\bar{\phi }_{i} - \phi_{ - } } \right)} \mathord{\left/ {\vphantom {{\left( {\bar{\phi }_{i} - \phi_{ - } } \right)} {\left( {\sqrt 2 \sigma_{i} } \right)}}} \right. \kern-0pt} {\left( {\sqrt 2 \sigma_{i} } \right)}}} \right], \\ r_{\text{I}}^{\text{o}} \left( {\phi_{\text{o}} ,\sigma_{\text{o}} } \right) & = \tfrac{1}{2}\,{\text{erfc}}\left[ {{{\left( {\phi_{\text{o}} - \phi_{ - } } \right)} \mathord{\left/ {\vphantom {{\left( {\phi_{\text{o}} - \phi_{ - } } \right)} {\left( {\sqrt 2 \sigma_{\text{o}} } \right)}}} \right. \kern-0pt} {\left( {\sqrt 2 \sigma_{\text{o}} } \right)}}} \right]. \\ \end{aligned} $$

The arguments of these functions were calculated in the pair approximation as defined in Geeves et al. (2011), Smith (2001), and Smith and Geeves (2003), so $\bar{\phi }_{i} = \bar{\phi } (x_{i} ,y_{i} )$, and *σ*_*i*_ = *σ*_*ϕ*_ (*x*_*i*_, *y*_*i*_) for pinning sites at distances *x*_*i*_ and *y*_*i*_ on either side of the site *i* in segment *m*. Along each CFC, a sequence of segments *I*_*m*_ is formed between any two nearest-neighboring pinning sites, where *m* denotes the currently considered segment. The discrete functions $\bar{\phi }_{i} = \bar{\phi } (x_{i} ,y_{i} )$, and *σ*_*i*_ = *σ*_*ϕ*_ (*x*_*i*_, *y*_*i*_) take different forms according to whether the pinning sites at the segment *m* ends are both generated by bound myosin-S1s or by bound TnIs, or by one bound myosin-S1 and one bound TnI. Closed formulae for $\bar{\phi }_{i} = \bar{\phi } (x_{i} ,y_{i} )$ and *σ*_*i*_ = *σ*_*ϕ*_ (*x*_*i*_, *y*_*i*_) are derived from Smith (2001) and given in the Electronic Supplementary Material (appendix B) reported in Geeves et al. (2011). This approach may not strictly satisfy the detailed balance principle (Howard 2001; Onsager 1931a,b; Wegscheider 1911), but considering all the other uncertainties, we believe that the predicted overall kinetics are not significantly different from the ones assessed by more rigorous approaches (Geeves et al. 2011).

For S1 binding to actin, let *k*_M1_ be the rate constant for weak myosin-S1 binding to F-actin when the CFC may occupy a range of closed positions, *ϕ* ≥ *ϕ*_o_ and let *k*_M2_ be the state transition rate constant from weak to strong binding when the CFC is in a range of open positions, *ϕ* ≥ *ϕ*_+_. The rates of S1 binding to actin and the isomerization are weighted functions of the corresponding transition rates, $k_{\text{M1}}^{\text{o}}$ and $k_{\text{M2}}^{\text{o}}$, from the closed state.

5 $$ \begin{aligned} k_{{{\text{M}}1}} (s_{i} ) & = \frac{{r_{{{\text{M}}1}} \left( {\bar{\phi }_{i} ,\sigma_{i} } \right)}}{{r_{{{\text{M}}1}}^{\text{o}} \left( {\bar{\phi }_{\text{o}} ,\sigma_{\text{o}} } \right)}}k_{{{\text{M}}1}}^{\text{o}} , \\ k_{{{\text{M}}2}} (s_{i} ) & = \frac{{r_{{{\text{M}}2}} \left( {\bar{\phi }_{i} ,\sigma_{i} } \right)}}{{r_{{{\text{M}}2}}^{\text{o}} \left( {\bar{\phi }_{\text{o}} ,\sigma_{\text{o}} } \right)}}k_{{{\text{M}}2}}^{\text{o}}. \\ \end{aligned} $$

Here, the weighted functions are proportional to the fraction of time when these transitions are possible, i.e., to the probabilities that the CFC is at positions *ϕ* ≥ *ϕ*_o_ for S1 binding and at positions *ϕ* ≥ *ϕ*_+_ for isomerization:

6a $$ \begin{aligned} r_{{{\text{M}}1}} \left( {\bar{\phi }_{i} ,\sigma_{i} } \right) & = \tfrac{1}{2}{\,\text{erfc}}\left[ {{{\left( {\phi_{\text{o}} - \bar{\phi }_{i} } \right)} \mathord{\left/ {\vphantom {{\left( {\phi_{\text{o}} - \bar{\phi }_{i} } \right)} {\left( {\sqrt 2 \sigma_{i}} \right)}}} \right. \kern-0pt} {\left( {\sqrt 2 \sigma_{i} } \right)}}} \right] \\ r_{{{\text{M}}2}} \left( {\bar{\phi }_{i} ,\sigma_{i} } \right) & = \tfrac{1}{2}{\,\text{erfc}}\left[ {{{\left( {\phi_{ + } - \bar{\phi }_{i} } \right)} \mathord{\left/ {\vphantom {{\left( {\phi_{ + } - \bar{\phi }_{i} } \right)} {\left( {\sqrt 2 \sigma_{i}} \right)}}} \right. \kern-0pt} {\left( {\sqrt 2 \sigma_{i} } \right)}}} \right] \\ \end{aligned} $$

and

6b $$ \begin{aligned} r_{{{\text{M}}1}}^{\text{o}} \left( {\bar{\phi }_{\text{o}} ,\sigma_{\text{o}} } \right) & = \tfrac{1}{2} \\ r_{\text{M2}}^{\text{o}} \left( {\bar{\phi }_{\text{o}} ,\sigma_{\text{o}} } \right) & = \tfrac{1}{2}{\,\text{erfc}}\left[ {{{\left( {\phi_{ + } - \bar{\phi }_{\text{o}} } \right)} \mathord{\left/ {\vphantom {{\left( {\phi_{ + } - \bar{\phi }_{\text{o}} } \right)} {\left( {\sqrt 2 \sigma_{{\phi_{\text{o}} }} } \right)}}} \right. \kern-0pt} {\left( {\sqrt 2 \sigma_{{{\text{o}} }} } \right)}}} \right]. \\ \end{aligned} $$

The functions $r_{{{\text{M}}1}} \left( {\bar{\phi }_{i} ,\sigma_{i} } \right)$ and $r_{{{\text{M}}2}} \left( {\bar{\phi }_{i} ,\sigma_{i} } \right)$ are also the cumulative forms of Gaussian distributions (Smith 2001). Thus, the rates $k_{\text{M1}}^{\text{o}}$ and $k_{\text{M2}}^{\text{o}}$ are reduced by the factors *r*_M1_ and *r*_M2_ (and also renormalized by $r_{{{\text{M}}1}}^{\text{o}}$ and $r_{{{\text{M}}2}}^{\text{o}}$) to provide the effective rates when the CFC lies in the angular range where the state transitions are permitted. For other CFC configurations, however, the binding rates are set to zero.

### Monte Carlo simulations

We employed the standard Metropolis algorithm where a kinetic transition in time Δ*t* is implemented when a random number in (0, 1) lies in the range (0, *k*∆*t*), where *k* is the first-order transition rate constant. This algorithm generates a Markov process if $k\Updelta t \ll 1$, so that at most one transition occurs per Monte Carlo time step in a single subsystem. Thus, ∆*t* must be less than the inverse of the fastest rate constant of the system, and in practice $k{\kern 1pt} \Updelta t \le 0.001$ was required to achieve satisfactory statistics. The transition rates between actin–myosin states are applied to *N* actin sites per CFC, i.e., per strand of an actin filament. The states of all *N* sites, whether free, occupied by weakly bound or strongly bound myosin, are listed by occupation numbers which are updated at each Monte Carlo time step. In separate drawings, TnI–actin transitions are updated for every seventh actin site, i.e., at the actin sites where TnI can bind. The subsystem studied in this way is defined by one strand of a single actin double helix, for which *N* = 700 and the number of TnI-actin binding sites is *I* = *N*/7 = 100. The subsystem corresponds to the actin filament length of 3.85 μm. Repeated Monte Carlo simulations include runs over large numbers of filaments with two strands of the actin double helix per filament in order to provide uniform sampling of the dynamics present in a solution experiment. The possible interactions between the CFCs on the same actin filament are neglected. Although Lehman et al. showed evidence that the Tm chains on two different strands may interact via Tn (Galinska-Rakoczy et al. 2008), we believe that these interactions may not significantly affect our analysis.

**Fig. 3:**
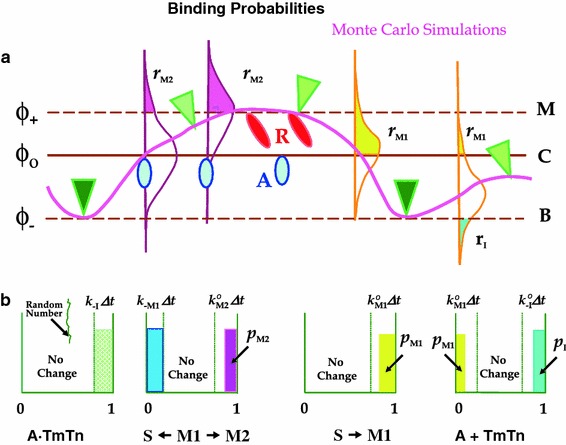
Monte Carlo simulations: construction of state transition probabilities. **a** The mean position of the CFC, $\bar{\phi }$, along an actin filament (*pink line*) for the current position of bound TnIs (*green filled triangles*), and bound myosin-S1s in A-state (*blue ovals*) and R-state (*red ovals*). Unbound TnIs are depicted as *light-green filled triangles*. The chain is pinned at locations of bound TnI and myosin-S1 and can move azimuthally between these sites on the actin filament surface. The CFC fluctuations are represented by a Gaussian distribution at each actin site along an actin filament strand (Fig. 2), and here only a few representative examples are shown. Myosin binding to A-state is possible for angles *ϕ* ≥ *ϕ*_o_, and the R-state for angles *ϕ* ≥ *ϕ*_+_. The rebinding of TnI, and therefore pinning of the CFC to angle *ϕ*_−_, is possible for *ϕ* ≤ *ϕ*_−_ (i.e., for $\left| \phi \right| \ge \left| {\phi_{ - } } \right|$ and *ϕ* < 0). For simplicity we set *ϕ*_o_ = 0. **b** The state transition probabilities from time *t* to time *t* + Δ*t* are represented by probability intervals (bins) at each actin site. Within a Monte Carlo step, Δ*t*, the probability in the range from 0 to 1 is divided into probability intervals where the transitions may occur and an interval where the current state will not change, denoted “no change.” The state transition probabilities, *p*_tr_, are typically equal to *k*Δ*t*, but some binding transitions are further inhibited by fluctuating CFC positions which prohibit S1 binding to actin for a fraction of the time step Δ*t*. The fraction of Δ*t* when the transition can occur is denoted as *r*_I_ for TnI binding to actin, and as *r*_M1_ and *r*_M2_ for myosin binding and isomerization, respectively. The transition will occur only if the drawn random number is within the transition bin values, *p*_I_, *p*_−I_, *p*_M1_, *p*_−M1_, *p*_M2_, and *p*_−M2_. These probabilities are modulated by weight factors ${{r_{\text{I}} } \mathord{\left/ {\vphantom {{r_{\text{I}} } {r_{\text{I}}^{\text{o}} }}} \right. \kern-0pt} {r_{\text{I}}^{\text{o}} }}$, ${{r_{\text{M1}} } \mathord{\left/ {\vphantom {{r_{\text{M1}} } {r_{\text{M1}}^{\text{o}} }}} \right. \kern-0pt} {r_{\text{M1}}^{\text{o}} }}$, and ${{r_{\text{M2}} } \mathord{\left/ {\vphantom {{r_{\text{M2}} } {r_{\text{M2}}^{\text{o}} }}} \right. \kern-0pt} {r_{\text{M2}}^{\text{o}} }}$ to take into account the current position of the CFC versus the transition rates from closed state and corresponding CFC. Heretofore, the inhibition factors *r* reduce the ordinary binding transition rates in proportion to the availability of the binding sites (Eq. 5) or define weight factors for modulation of TnI rebinding depending on the position of the CFC in the current configuration (Eq. 3). At each Monte Carlo step we perform two drawings: first for binding or detachment of TnI over all TnI actin binding sites (*far left* and *far right bins*), and the second which defines transitions between actin-S1 states (two *middle bins*)

It is assumed that the thermal motions follow a normal distribution around the CFC mean position $\bar{\phi }_{i}$ with a standard deviation *σ*_*i*_, and the values *r*_M1_ and *r*_M2_ specify the fraction of the time interval during which the CFC will permit weak and strong myosin-S1 binding to actin. The weight factor $r_{\text{I}} /r_{\text{I}}^{\text{o}}$ modulates the TnI rebinding rate of an unrestricted CFC, $k_{\text{I}}^{\text{o}}$ at $\bar{\phi } = \phi_{\text{o}}$ , to the actual TnI rebinding rate, *k*_I_, for a chain at an arbitrary position $\bar{\phi } \ne \phi_{\text{o}}$ at the current configuration of a CFC. The factors *r*_M1_, *r*_M2_, and $r_{\text{I}} /r_{\text{I}}^{\text{o}}$ are used in defining the Monte Carlo state transition probabilities (Fig. 3). At each Monte Carlo step, the state transition probabilities from the current state are calculated from the corresponding effective state transition rates and the duration of the time step. The transition rate constants include: detachment of and rebinding of TnI to actin, *k*_−I_ and *k*_I_, myosin binding and detachment, *k*_M1_ and *k*_−M1_, and isomerization forward and backward rate constants, *k*_M2_ and *k*_−M2_. Some of these rate constants are inhibited by the position of the fluctuating CFC. We separate two processes: (1) release and rebinding of TnI to actin, which is restricted to actin–TnI sites, and (2) myosin binding and isomerization, which can occur at any of the myosin binding sites on F-actin. The first Monte Carlo drawing, i.e., randomly chosen number, defines whether the TnI detaches from actin or rebinds back after the detachment via prior defined probabilities. The probability of the release of TnI, which permits azimuthal movement of the Tm chain on the actin surface upon binding of Ca^2+^ to actin, is $p_{{ - {\text{I}}}} = k_{{ - {\text{I}}}} \Updelta t$, and the probability of TnI rebinding to actin is $p_{\text{I}} = \left( {{{r_{\text{I}} } \mathord{\left/ {\vphantom {{r_{\text{I}} } {r_{\text{I}}^{\text{o}} }}} \right. \kern-0pt} {r_{\text{I}}^{\text{o}} }}} \right)k_{\text{I}}^{\text{o}} \Updelta t$. The second Monte Carlo drawing defines transitions between actin and myosin-S1 states. Myosin weak binding to actin has a probability of $p_{\text{M1}} = \delta \cdot r_{\text{M1}} k_{{{\text{M}}1}} \Updelta t$, and myosin detachment has a probability of $p_{{ - {\text{M1}}}} = k_{{ - {\text{M1}}}} \Updelta t$. The transition probability from weak to strong binding is $p_{\text{M2}} = r_{\text{M2}} k_{\text{M2}} \Updelta t$, and the backward transition is $p_{{ - {\text{M2}}}} = k_{{ - {\text{M2}}}} \Updelta t$. The time step used is sufficiently small that the probability that these transitions will occur in the current step is smaller than 0.1 % per CFC in order to ensure satisfactory statistics.

The parameter *δ* defines the negative cooperativity affecting inhibition of the myosin binding rate, which is presumably caused by a steric hindrance imposed by previously bound myosin-S1s. The process of crowding is especially important at higher occupancy of actin sites (>50 %) when considerable slowing of myosin-S1 binding is observed. Computational tests showed that reasonably good fits of excess myosin binding isotherms can be achieved by introducing a parameter *δ* defined as

7 $$ \delta = 1 - 0.82\left( {{{n_{\text{AM}} } \mathord{\left/ {\vphantom {{n_{\text{AM}} } {n_{\text{A}} }}} \right. \kern-0pt} {n_{\text{A}} }}} \right), $$

where $n_{\text{AM}}$ is the number of actin sites on a filament with a bound myosin head (i.e., either in state weak or strong state) and $n_{\text{A}}$ is the total number of actin sites on a filament strand (usually 700). This enables the model to match the experimental data well. It leads to a linear slowing of the myosin binding by up to 1/5 of the original binding rate. Equation 7 is only a convenient empirical formula, and this process can be modeled more precisely when data for unregulated actin are available. In a recently published paper, a steric model of this slowing was incorporated into Monte Carlo simulations by reducing the rate of myosin binding by a factor, determined by fitting the high-calcium transient, for each occupied nearest-neighbor site (Geeves et al. 2011). This simulation provided reasonable, but not excellent, fits of the stopped-flow transients, suggesting that steric modeling should include additional factors that can be incorporated into future studies.

### Model parameters

The mechanical parameters *κ* and *α* of the unpinned CFC were estimated as follows (see also Table 1). The angular CFC bending stiffness per unit length (Eq. 1) is calculated as $\kappa = \kappa_{\text{Tm}} R^{2}$ from the bending stiffness *κ*_Tm_ of tropomyosin. The stiffness *κ*_Tm_ is estimated from measurements of the persistence length $L_{{p{\text{Tm}}}} = \kappa_{\text{Tm}} /k_{\text{B}} T$ of the tropomyosin chain in solution (Phillips and Chacko 1996; Sousa et al. 2010). Here, *R* is the radius at which tropomyosin sits on the actin filament, *k*_B_ is Boltzmann’s constant, and *T* is absolute temperature. Because Tn bound to the Tm chain increases the stiffness of the Tm chain in calculations of *κ*_Tm_ (denoted as $k_{\text{TmTn}}$ ) and *κ*, we use a persistence length of a Tm–Tn chain, $L_{{p{\text{TmTn}}}}$, rather than of a Tm chain alone (Table 1). Values of the pinning angles *ϕ*_±_ were estimated from cryo-EM reconstructions (Poole et al. 2006; Vibert et al. 1997). The standard deviation $\sigma_{\text{o}} = \left( {k_{\text{B}} T/8\kappa \xi^{3} } \right)^{{{1 \mathord{\left/ {\vphantom {1 2}} \right. \kern-0pt} 2}}}$ of the free CFC was estimated from the ratio of blocked states to open/closed states, using a normal distribution of CFC angles. For skeletal A–Tm–Tn at high calcium in the absence of myosin, Pirani et al. (2005) observed that 20 % of tropomyosin segments were at angular position *ϕ* ≤ *ϕ*_−_, corresponding to the blocked state. The cumulative probability, $r_{\text{I}} = \Upphi \left( {\phi_{ - } ,\sigma_{\text{o}} } \right) = 0.2$ ensures that 20 % of the chain is in the blocked position, giving $\sigma_{\text{o}} = {{\phi_{ - } } \mathord{\left/ {\vphantom {{\phi_{ - } } {( - 0.84162)}}} \right. \kern-0pt} {( - 0.84162)}},$ where $\Upphi^{ - 1} \left( {0.2,1} \right) = - 0. 8 4 1 6 2$. Thus, for *ϕ*_−_ = 25° (Poole et al. 2006; Vibert et al. 1997), *σ*_o_ = 29.7°. Hence, the persistence length of the confined chain, 1/*ξ*, is estimated to be 22.2 nm. The degree of azimuthal confinement follows from the identity *α* = 4*κξ* ^4^.

Table 1 also lists rate constants for the unregulated transitions and optimum rate constants for the regulated ones. The second-order myosin binding rate constant allows simulations to be made at different concentrations of myosin-S1, chosen to match the experimental data with excess myosin or excess actin ([S1]/[A] = 10 or 0.1) (Boussouf et al. 2007a, b). To allow for the progressive depletion of free myosin-S1 during binding, *k*_M1_ is proportional to [M] = [M_tot_] − [AM] = [M_tot_] − *f*[A_tot_], where *f* is the fraction of actin sites occupied by myosin. Here, [AM] denotes all actomyosin states; i.e., it includes both weakly and strongly bound myosin states. The probability of detaching the bound myosin is assumed to be constant. In all simulations, the value of ∆*t* was set at 10^−5^ s; the number of CFCs was 2,000, corresponding to 1,000 actin filaments, for excess actin and 200 (i.e., 100 actin filaments) for excess myosin-S1. Although filaments of various lengths were present in the solution experiments, the simulation results were not sensitive to filament length. For simplicity we assumed 100 TnTm units per actin filament strand, i.e., 700 actin monomers, corresponding to actin filament length of 3.85 μm.

### Assessment of Ca^2+^ parameters from two sets of stopped-flow data

The model predictions were tested against measurements of the fluorescence intensity of pyrene-labeled actin during kinetic (stopped-flow) experiments. These experiments have been described in detail in published work (Boussouf et al. 2007a, b; Boussouf and Geeves 2007), and the data used here were reported in Mijailovich et al. (2012). We will briefly here describe the experimental methods used. The proteins were all prepared from rabbit fast skeletal muscle. Myosin-S1 was prepared by chymotryptic digestion of myosin and column purified to isolate the A1 light chain isoform. Actin was prepared from a muscle acetone powder (Spudich and Watt 1971) and was labeled with pyrene to >90 % at Cys374 as described by Criddle et al. (1985). A Tm–Tn complex was isolated from the same acetone powder used for the actin preparation as described by Ebashi et al. (1971) and Greaser and Gergely (1971).

In stopped-flow experiments, fluorescently labeled (regulated) actin filaments in solution are first equilibrated at a specific Ca^2+^ concentration and then mixed with myosin-S1. The change in pyrene fluorescence (excitation at 365 nm, emission at 405 nm) was recorded at each calcium concentration, and we collected two sets of data: one for excess myosin ([S1]/[A] = 10) and the other for excess actin ([S1]/[A] = 0.1). All data were collected at 20 °C in a buffer containing 100 mM KCl, 5 mM MgCl_2_, and 20 mM 3-(N-Morpholino)propanesulfonic acid (MOPS) pH 7.0. The calcium concentration was controlled by the addition of ethylene glycol tetraacetic acid (EGTA) and calcium-saturated EGTA at 2 mM to give the required free calcium concentration.

### Procedure for estimation of CFC model parameters

The model predictions were tested against measurements of the pyrene fluorescence intensity during the stopped-flow experiments described above. In these experiments a drop in pyrene fluorescence is proportional to myosin binding to actin in the R-state. Thus, the calculated instantaneous fractions of actin sites that are not occupied or which are in the A-state (i.e., which are not in the R-state), denoted ${\mathbf{g}}({\varvec{\lambda}},t)$, can be compared with corresponding experimental data, ${\mathbf{d}}^{\text{obs}} (t)$, at the same instant. Here, the vector ${\ddot{\mathbf{e}}} = \left({\lambda_{1}, \ldots,\lambda_{m}} \right)$ represents the set of *m* free model parameters that need to be estimated (Mijailovich et al. 2010).

By fitting sets of the stopped-flow data for excess myosin or excess actin ([S1]/[A] = 10 or 0.1) for a range of Ca^2+^ concentrations, we estimated a single free parameter, the TnI detachment rate constant *k*_−I_, as a function of Ca^2+^ concentration and the [S1]/[A] ratio. Establishing the dependence of the equilibrium constant *K*_I_ on Ca^2+^ concentration in solution could serve as a basis for developing models of thin filament regulation in muscle fibers.

## Results

The predicted time courses of myosin binding to regulated actin filaments show evidence of cooperativity, in much the same way that titration data do for the equilibrium fraction of myosin-occupied sites (Maytum et al. 1999). The initial binding response is relatively slow because binding is inhibited by the Tm chain, and is then accelerated by increased accessibility of opened actin binding sites by the shift of Tm-Tn chain in the neighborhood of the first bound myosin. This myosin–myosin cooperativity is most evident at low calcium with a high concentration of myosin-S1.

**Fig. 4:**
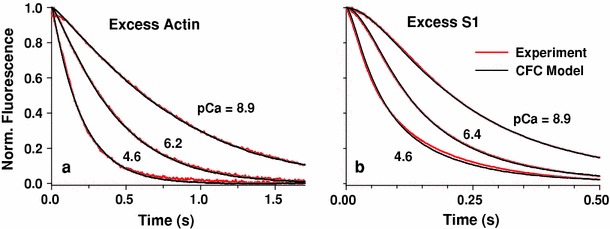
Best fits of the CFC model (*black lines*) to stopped-flow data (*red lines*) of myosin-S1 binding to actin in solution at high, low, and an intermediate concentration of Ca^2+^. **a** Excess-actin concentration ([actin] = 2.5 μM, [S1] = 0.25 μM). The estimated *K*_B_ values for pCa of 8.9, 6.2, and 4.6 are 0.12, 0.47, and 11.89, respectively. **b** Excess myosin-S1 concentration. ([actin] = 0.5 μM, [S1] = 5.0 μM). The estimated *K*_B_ values for pCa of 8.9, 6.4, and 4.6 are 0.11, 0.36, and 11.89, respectively. The parameters used in the simulations are given in Table 1

With excess actin, the observed binding transients at different calcium levels were all fitted by Monte Carlo simulations in which the only varied parameter was the detachment rate of TnI from actin, *k*_−I_, which strongly depends on Ca^2+^ concentration (Fig. 4). The one-parameter fit was possible because unregulated or optimal myosin rates were carefully chosen to match equivalent titration data and/or transient data at high calcium. The precise value of the reattachment rate, *k*_I_, is not important because TnI is in rapid equilibrium with actin on the time scale of myosin binding, so data fitting is controlled by the equilibrium constant $K_{\text{I}} \equiv k_{\text{I}} /k_{{ - {\text{I}}}}$. We chose to use *K*_I_ as an adjustable fitting parameter, following the McKillop–Geeves approach where the key regulated constant is $K_{\text{B}} \equiv 1/K_{\text{I}}$. For simplicity we estimated a single model parameter, *K*_B_, using the same notation as reported in Mijailovich et al. (2012), rather than predict the calcium dependence of *K*_I_ from an allosteric model of troponin–calcium interactions (Smith and Geeves 2003), in order to avoid nonuniqueness of the estimated multiple parameters (Mijailovich et al. 2010).

**Fig. 5:**
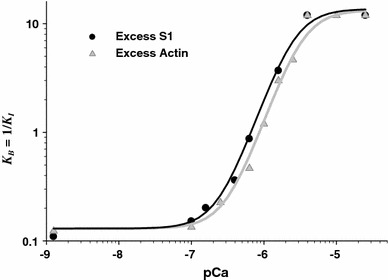
Calcium dependence of the equilibrium rate constant of Tn unbinding and rebinding $K_{\text{B}} = 1/K_{\text{I}} = {{k_{{ - {\text{I}}}} } \mathord{\left/ {\vphantom {{k_{{ - {\text{I}}}} } {k_{\text{I}} }}} \right. \kern-0pt} {k_{\text{I}} }}$. Both excess actin (*gray line and symbols*) and excess actin (*black line and symbols*) show a sigmoidal *K*_B_ dependence on pCa with Hill coefficient of 2.2 for excess actin and 2.1 for excess S1

Having matched model predictions to the experimental transients at different calcium levels, the equilibrium constant $K_{\text{B}} \equiv 1/K_{\text{I}}$ is obtained. Figure 5 shows that $K_{\text{B}} \left( {\left[ {\rm Ca} \right]} \right) \equiv 1/K_{\text{I}}$ exhibits a sigmoidal curve, and the curves for the excess-myosin and excess-actin data are almost in exact agreement, as expected if *K*_I_ is derived from the same allosteric model of calcium–TnI–actin interactions. These curves have a Hill coefficient of 2.2 for excess actin and 2.1 for excess S1, slightly higher than the value of 1.8 obtained from solution measurements of the rate of myosin binding to fully regulated actin as a function of calcium (Head et al. 1995) for skeletal TnC. In an earlier study of the CFC model, this modest cooperativity was generated by the CFC, which requires that $\phi_{ - } \ne 0$ (Smith and Geeves 2003). Thus, the Hill coefficients obtained here appear to be consistent with previous findings.

**Fig. 6:**
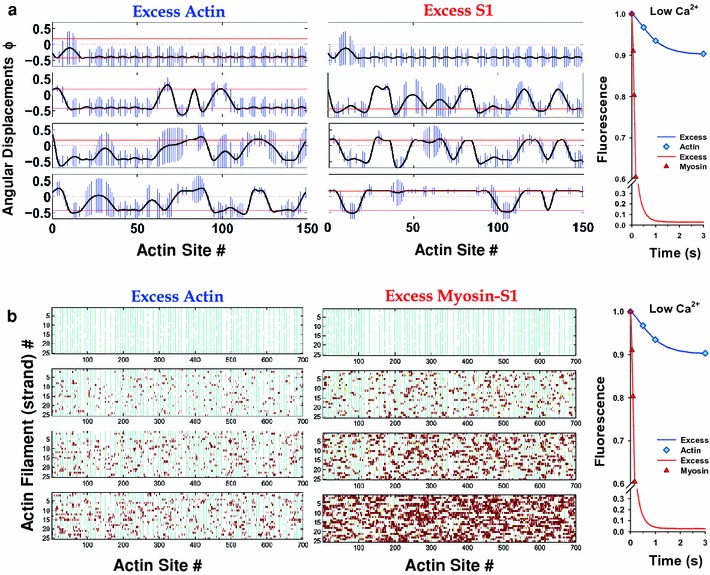
**a** Angular displacements, *ϕ*_−_, along a strand of an actin filament at low calcium (pCa = 8.9 case) at four time points of a stopped-flow transient for excess actin and for excess myosin. Only a part of the whole strand associated with CFC is shown (150 actin monomers). The *bars* represent standard deviation of CFC position due to thermal fluctuation. Four characteristic plots for four different times for each excess actin and excess myosin-S1 are shown. The time points at which graphs are sequentially collected are shown at pyrine fluorescence transients (*right*). In absence of myosin-S1, most of the CFC is pinned at every seventh actin binding site at angular position *ϕ*_−_ where CFC fluctuations are equal to zero. Strongly bound myosin favors binding of myosin to neighboring actin sites, forming long patches of the CFC in the open position, *ϕ*_+_, where bound myosins cluster, and patches of CFC in the blocked position, *ϕ*_−_. **b** The distribution of troponin–actin and myosin–S1-actin states during stopped-flow transients at low calcium (pCa = 8.9) for excess actin and for excess myosin. Four characteristic plots for four different times are shown for excess actin and for excess myosin-S1. The time points at which graphs are sequentially collected are shown at pyrine fluorescence transients (*right*). Each *row* represents a strand of an actin filament displaying 26 strands, i.e., 13 actin filaments. Each actin strand contains 700 monomers and 100 TnI binding sites. Unoccupied actin sites are *white*, troponin bound sites are denoted by *cyan bars*, weakly bound myosin-S1 as *yellow bars*, and strongly bound myosin-S1 as *dark-red bars*. The clusters of strongly bound myosin-S1 are clearly visible

The Monte Carlo method provides an explicit realization of the molecular state of the regulated filament at each time step, which is instructive for understanding how the model operates in detail. Figure 6a shows the evolution of CFC conformations at low calcium for both excess actin and excess S1. At early times, a few myosins bind under a chain initially pinned at nearly all TnI–actin sites (blocked states). In most cases, myosin binding forces the detachment of the nearest TnIs, shifts CFC toward larger *ϕ* values, and permits additional myosin binding. Also, bound myosin prevents those detached TnIs from reattaching. As the steady state is approached, clusters of bound myosins form under a single kink of the bent chain. With excess myosin (Fig. 6a, middle panel), these processes are accelerated in time and the final state is one in which the filament is nearly saturated with myosin, where the chain is pinned primarily in the open position with only a few isolated regions where the chain is pinned by bound TnIs. Plots of site occupancies, at the same four time points on 13 actin filaments (i.e., 26 strands), with each row representing a strand of an actin filament, are shown in Fig. 6b; clustering is clearly evident at early times of the excess-myosin simulation when occupancy of actin sites is less than 50 %. In both cases, when clustering is present, the size of the regulatory unit is larger than the structural unit of seven actin monomers.

**Fig. 7:**
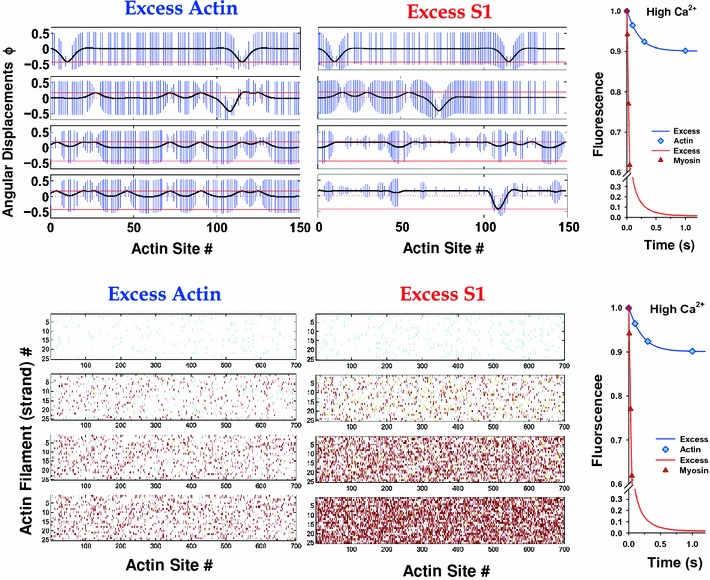
**a** Angular displacements along a strand of an actin filament at high calcium (pCa = 4.6) at four time points of stopped-flow transients for excess actin and excess myosin. The plots are displayed in the same way as in Fig. 6a. In absence of myosin-S1 only sparse bound TnI keeps the CFC pinned at *ϕ*_−_, while the majority of the CFC length sits at closed position at *ϕ*_o_ = 0. In presence of myosin-S1, strongly bound myosin pushes CFC to open position, *ϕ*_+_. **b** The distribution of troponin–actin and myosin-S1 states during stopped-flow transients at high calcium (pCa = 4.6) and for both excess actin and excess myosin. The plots are displayed in the same way as in Fig. 6b. Because of sparse pinning of the CFC by TnI, the CFC is mostly in the closed and open states, permitting random binding of myosin-S1; consequently, clustering of bound myosin-S1 is not observed

At high calcium, the picture is primarily one of myosin-based cooperativity and clustering (Fig. 7). Now the initial state is mainly that of the free chain in angular position at the minimum of the confining potential, *ϕ*_o_, analogous to the closed state of the McKillop–Geeves model, although two instances of bound TnIs are present in the excess-actin simulation shown. Both these blocked regions disappear as myosin binding proceeds, demonstrating that myosin is seven times more powerful than TnI in moving the chain through sheer force of numbers, even if their affinities are about the same. With excess myosin (Fig. 7a, middle panel), the filament saturates with myosin more quickly, giving holes of closed state in a line of open states. However, TnI can still bind weakly to actin and has taken the opportunity to do so (see the bottom right-hand graph in Fig. 7a) where there is a comparative absence of bound myosins. The site occupancies (Fig. 7b) show a much more random distribution of bound myosin-S1 than at low Ca^2+^ even in early stages of the binding process, suggesting that at high calcium most of the regulatory units are open and clusters of bound myosin-S1 appear sparsely distributed, especially in the case of excess myosin.

## Discussion

The spatially explicit model of thin filament regulation developed here, based on the theoretical work of Smith et al. (Geeves et al. 2011; Smith 2001; Smith and Geeves 2003; Smith et al. 2003), provides a structurally based rather than empirically based description of the cooperative effects of myosin binding to regulated actin in solution. The key advance in the current work is the introduction of a CFC (Smith et al. 2003) instead of the widely used, seven actin monomer long, TmTn rigid units with or without empirically induced cooperativity between the neighboring units (Chen et al. 2001; Hill et al. 1980; McKillop and Geeves 1993; Mijailovich et al. 2012). Extending the approach of Smith et al. (Smith 2001; Smith and Geeves 2003; Smith et al. 2003), we developed a Monte Carlo algorithm to quantify spatially explicit myosin-S1 binding to regulated F-actin in solution. By fitting the model predictions to a set of stopped-flow transients for a large range of Ca^2+^ concentrations, we determined the Ca^2+^ dependence of the equilibrium rate constant of TnI binding to actin, $K_{\text{I}}$. We found an almost identical sigmoidal relationship between $K_{\text{B}} \equiv 1/K_{\text{I}}$ and pCa for both excess-actin to myosin concentration and excess-myosin to actin concentration. This finding is important because it confirms previous notions that myosin binding per se is only weakly dependent, or perhaps not dependent at all, on the Ca^2+^ concentration. The predicted Hill coefficient $K_{\text{B}} \equiv 1/K_{\text{I}}$ is slightly larger than 2, confirming a significant level of cooperative switching in the TmTn units and slightly above that required by two calcium ions binding a TnC. The model also predicted a size for the cooperative unit of myosin binding of 8.2 actin monomers, which is larger than the structural unit of 7. This is interesting because a unit size larger than 7 is likely to be a consequence of chain flexibility rather than weak TnI–TnI cooperativity. At lower concentrations of Ca^2+^, the model predicted clustering of bound myosin-S1s in sparsely distributed open regions. These predictions are consistent with the findings of Vibert et al. (1997). Finally, a simple empirical relationship which defines the slowing of myosin binding at high occupancy of actin sites gave good fits to the myosin binding transients at all Ca^2+^ concentrations.

For easy comparison with other models of thin filament regulation (Chen et al. 2001; Geeves et al. 2011; Hill et al. 1980; Maytum et al. 1999; McKillop and Geeves 1993; Mijailovich et al. 2010; Mijailovich et al. 2012), we plot in Fig. 5 the $K_{\text{B}} \equiv 1/K_{\text{I}}$–pCa relationship rather than the *K*_I_–pCa relationship. For illustration, in the Electronic Supplementary Material we show the best fits of our model compared with the best fits achieved by the Hill model as formulated by Chen et al. (2001) and the McKillop–Geeves model (1993) as formulated by Mijailovich et al. (2012) to the same dataset (Supplementary Fig. S1). These plots confirm that all three models are capable of achieving equally excellent fits to the data. Furthermore, estimated *K*_B_–pCa relationships of CFC and McKillop–Geeves model were similar too (Supplementary Fig. S2). Although the comparisons with independent-regulatory-unit models such as the McKillop–Geeves model (McKillop and Geeves 1993) are instructive, the conceptual bases of these models are quite different from the CFC model. The quantity 1/*K*_I_ is loosely analogous to the quantity *K*_B_ of the independent-unit model of McKillop and Geeves, which specifies the equilibrium between blocked and closed states. However, there are important differences. *K*_I_ is the equilibrium constant for the calcium-dependent detachment of TnI from actin, which is specific to these two proteins and does include the full spectrum of chain configurations, whereas *K*_B_ in the McKillop–Geeves model is the ratio of the populations of blocked and closed states defined by specific orientations of Tm.

In order to avoid nonuniqueness of the estimated multiple model parameters, we reduced the model to a single free parameter, *k*_−I_, while all other parameters are held constant over all concentration of Ca^2+^ and with two actin-to-myosin concentration ratios. The overall good fits are provided by reasonable assessment of other model parameters from structural, biophysical, and biochemical data; For example, the tropomyosin pinning angle, *ϕ*_−_, and myosin-imposed Tm angular displacement, *ϕ*_+_, are taken from Poole et al. (2006) and Vibert et al. (1997), and the chain persistence length from Phillips and Chacko (1996) and Sousa et al. (2010), and as such they are not free parameters. In addition, the confining potential is derived from the experimental data of Pirani et al. (2005). Similarly, the rate constants for myosin binding, *k*_M1_, and isomerization, *k*_M2_, are chosen as the best fit of the myosin binding transients. The equilibrium constant $K_{\text{M2}} = 100$ we used here is slightly smaller than normally quoted $K_{\text{M2}} = 200$ (Maytum et al. 1999), and it is taken for convenience to better fit the data. Although all these parameters could vary over a range of values, when brought together, they gave a consistent set of parameters that provided good fits to multiple sets of data.

The resulting sigmoidal $K_{\text{B}} \equiv 1/K_{\text{I}}$–pCa relationship predicts an apparent Hill coefficient of about 2.15, which is similar to that predicted by the McKillop–Gevees model of 2.07 (Mijailovich et al. 2010). Note that a value of the Hill coefficient for the calcium switch of TnC that is less than the number of binding calcium sites (i.e., <2) can be considered independent (Head et al. 1995).^2^ The Hill coefficient for calcium binding in fibers is reported to have values anywhere from 2 to 7 (Gordon et al. 2000), but this is measuring a different parameter than *K*_B_ and is associated with the force generated by strongly attached myosin bridges. In fibers, Moss et al. (1983) found a Hill coefficient of close to 2 at partial overlap of actin and myosin filaments, but they found a biphasic distribution for the Hill coefficient having value of 6.7 for pCa >6.5 and close to 2 for pCa <6.5 at full filament overlap. This increase in the cooperativity in solution and in fibers with added myosin in presence of calcium is consistent with evidence for myosin–TnI interactions reported by Bremel and Weber (1972) and Grabarek et al. (1983).

All CFC model parameters, except $K_{\text{B}} \equiv 1/K_{\text{I}}$, are assumed to be independent of calcium concentration and consistent with the parameter values of the McKillop–Geeves model used in several of our publications (McKillop and Geeves 1993; Mijailovich et al. 2010; Mijailovich et al. 2012). The sigmoidal $K_{\text{B}} \equiv 1/K_{\text{I}}$–pCa relationship in Fig. 5 is similar to the *K*_B_–pCa relationship obtained from the fits of the same stopped-flow data by the McKillop–Geeves model, reported by Mijailovich et al. (2012) (see Supplementary Fig. S2). The differences between these $K_{\text{B}} \equiv 1/K_{\text{I}}$–pCa relationships can be attributed to intrinsic differences between the McKillop–Geeves model and CFC model, because two McKillop–Geeves model parameters, namely *K*_T_ and the positive cooperativity factor, are replaced in the CFC model by the persistence length of the confined CFC, 1/*ξ*.

**Fig. 8:**
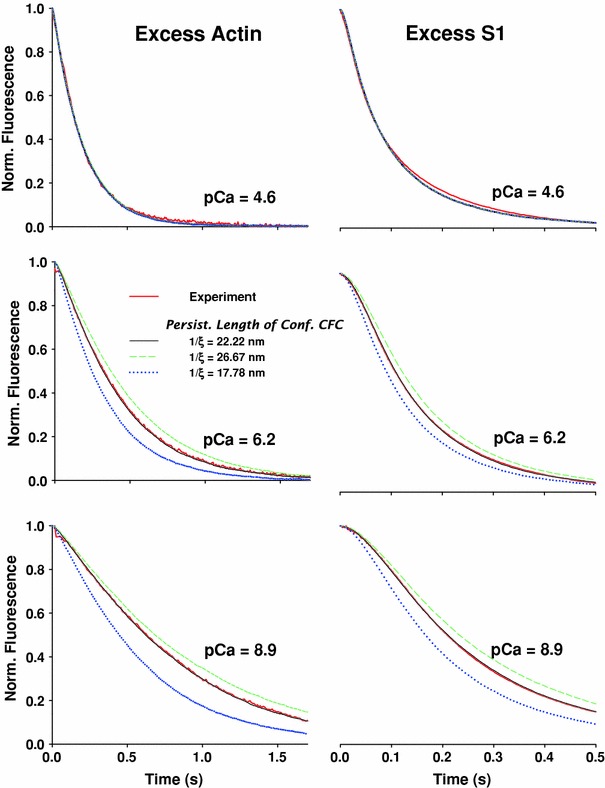
Effect of the persistence length of confined CFC, 1/*ξ*, on regulation of S1 binding. As shown in Fig. 4, the model predictions with persistence length of confined CFC of 1/*ξ* = 22.2 nm (*black solid lines*) closely follow the time course of normalized fluorescence (*red solid lines*) collected from stopped-flow experiments at three calcium concentrations. For both excess actin and excess S1, at high calcium concentration (pCa = 4.6), 20 % increase in 1/*ξ* (*green dashed line*) or 20 % decrease (*dotted blue line*) made almost no difference compared with the fit to the experimental data or the predictions with the original value 1/*ξ* = 22.2 nm. Decrease in calcium concentration resulted in significant departure of the predicted normalized fluorescence from both stopped-flow data and the fit with the original value 1/*ξ* = 22.2 nm. For this comparative analysis all other CFC model parameters were kept the same as displayed in Fig. 4. At medium and low calcium concentrations, the increase in 1/*ξ* to 26.67 nm decreases the predicted rate of decay of the normalized fluorescence compared with the experimental data, while reduction of 1/*ξ* to 17.78 nm increased the rate. The decrease of 1/*ξ* resulted in a much stronger departure from the experiments than the departure (in the opposite direction) for the equivalent increase in 1/*ξ*

The persistence length 1/*ξ* is the essential new parameter in the model, and in order to evaluate how the modeling results depend on the specific value used, we performed a sensitivity analysis of 1/*ξ*. In Fig. 8 the predicted fluorescence time courses are compared for a 20 % increase (green dashed lines) or 20 % decrease (blue dotted lines) in the originally value of 1/*ξ* = 22. 2 nm (black solid lines; data from Fig. 4).

As expected, at high calcium concentrations (pCa = 4.6), the transients were insensitive to a 20 % change in 1/*ξ*. This is because, at high calcium concentrations, the binding of S1 is not affected by the Tm–Tn CFC chain. At medium and lower calcium concentrations, an increase or decrease of 1/*ξ* showed slowed or accelerated fluorescence transients, respectively. Effectively, this means that an increase of 1/*ξ* will shift the *K*_B_–pCa curve to higher calcium concentrations due to the reduced angular standard deviation of free chain, *σ*_o_. Conversely, the smaller 1/*ξ* will shift the *K*_B_–pCa curve to lower calcium concentrations, due to increased *σ*_o_ and, therefore, increased sensitivity of *K*_B_ to calcium concentration.

At first sight a surprising result from the simulations with varying 1/*ξ* is the strong dependence of the fluorescence transient on calcium concentration for the excess-actin case. However, from the point of view of the CFC model, this behavior can be explained simply by associating the reduction of chain fluctuations with an increase in 1/*ξ*, and therefore a significant decrease in the availability of actin and a decrease of the effective rate constant of S1 binding (Eq. 5).

From Fig. 8 it is evident that, at medium and lower calcium concentration, the slope of the fastest part of the fluorescence decay, i.e., the middle of the curve, changes with an increase or decrease of 1/*ξ*, rather than a shift of the transient. Thus, refitting the data by varying *k*_−I_ will not be sufficient to provide a good fit to the data. The values of the equilibrium constant, *K*_I_, also become unacceptably large (i.e., *K*_B_=1/*K*_I_ small) at low calcium if only at low calcium if only *K*_I_ is changed (as shown in Geeves et al.  2011). This implies that both *K*_I_ and *k*_M1_ must be changed to give a good fit. This would require that *k*_M1_ has a strong dependence on calcium concentration, which is contrary to current thinking (Geeves et al. 2011; Mijailovich et al. 2010; Mijailovich et al. 2012). We therefore favor the simpler interpretation that the persistence length is well defined at 22.2 nm (Pirani et al. 2005) and that *K*_I_ is the only calcium-dependent parameter. This assertion provides a way to test the model.

Any change in the TnC used (different isoforms or mutations) would be expected to change the value of *K*_I_ but have little effect on the persistence length or the confining potential. Use of different Tm’s (isoforms or mutations) could alter the persistence length/confined potential but should not change *K*_−I_. Similarly, different myosins or myosin nucleotide complexes should change *k*_M1_ but should not alter the other two parameters. Note however that TnI or TnT interacts with both Tm and TnC and thus may affect either parameter. Note also that the model predictions in Fig. 8 show that a change in the persistence length of the confined chain, 1/*ξ*, can change the apparent calcium sensitivity of myosin binding because the apparent myosin binding rate constant is a function of both 1/*ξ* and *K*_I_.

In the current model, TnI–TnI and myosin–TnI interactions are only partially implemented by modulated rebinding of TnI to actin by the current configuration of the CFC. In contrast, the detachment of TnI from actin is assumed to be solely dependent on the Ca^2+^ concentration. The latter is true only if the TnI–actin energy well is sufficiently deep that any mechanical force in the absence of Ca^2+^ will be insufficient to perturb the bond from the stable equilibrium. In contrast, when one or two calcium ions are bound to TnC, i.e., when the affinity of TnI to actin is significantly reduced, a small perturbation (thermal or mechanical) will be sufficient to cause the detachment of TnI. In reality, this may not be always the case at low Ca^2+^, especially when rigor bridges displace TnI, as observed in low-Ca^2+^ myosin binding experiments, or at submaximal calcium concentrations, when such detachment also can be triggered by mechanical stretch (i.e., stretch activation; Pringle 1978). To date, no satisfactory molecular mechanism for stretch activation has been reported. While some degree of stretch activation occurs in all striated muscles, in vivo it functions primarily in muscles that contract rhythmically such as the indirect flight muscles (IFM) in many insects (Josephson et al. 2000) and vertebrate cardiac muscle, where it appears to aid cardiac ejection (Stelzer et al. 2006; Vemuri et al. 1999). Therefore, implementing strain-dependent detachment of TnI from actin is very important in studying diseases associated with dysfunction of Ca^2+^ regulation of muscle contraction. An important contribution in this direction has been recently reported (Geeves et al. 2011).

The kinetic version of the CFC model presented here has some limitations, which may require updating in the future. In particular, regulation by the CFC of the kinetics of myosin–actin and TnI–actin binding is assumed to be controlled solely by the statistics of the fluctuations of the CFC, which leaves open the question of whether the reverse rates are regulated. The CFC model shows that the corresponding affinities for actin are regulated in quite a different way, in terms of Gibbs energy differences between the states involved, so that it appears likely that the TnI rebinding rates will also be regulated quantities. A new approach is required which combines energetics with kinetics, and this problem is currently being addressed (Geeves et al. 2011). However, the good fits to transient data by the present model may not be significantly affected if the kinetically regulated transitions are predominantly irreversible; this condition is approximately satisfied except when the number of myosins bound to actin within a single TmTn unit is <1, which is only significant for early transients at low calcium. Because the mean position and fluctuation around that position of the CFC partially take into account strain-dependent rebinding rates of TnI to actin, the apparent kinetic errors in TnI rates will then have very little effect on myosin kinetics.

A modification to the myosin binding rate is necessary (Eq. 7) to fit the excess-myosin data, because with excess-myosin to actin concentration and fixed *k*_M1_ the model predicts faster myosin binding than is observed when more than 50 % of actin sites are occupied. We suggest that the slower experimental response is due to the reduced diffusion constant of free myosin-S1s in the vicinity of actin filaments heavily loaded with myosin. This phenomenon, specific to experiments in solution, needs further experimental and theoretical investigation.

In addition, the CFC model as originally proposed has several features which might need to be modified in the light of new experiments. Although there is new evidence for intermediate orientations of the chain and hence for bending compliance (Pirani et al. 2005), the chain of overlapping tropomyosins is obviously not a homogeneous elastic structure. It is not yet clear whether the chain is merely sterically hindered by a bound myosin (*ϕ* > *ϕ*_+_) or attracted to it, in which case *ϕ* = *ϕ*_+_. We used the latter for mathematical convenience in deriving formulae with the pair approximation. In practice, the consequences of these two possibilities would be quite similar, as the probability of CFC conformations at angles above *ϕ*_+_ is quite small if the distortion energy 4*κξ* ^3^ϕ_+_^2^ of the kink itself exceeds the thermal energy *k*_B_ *T*. It is also desirable to avoid the pair approximation, but that is a matter of convenience in manipulating the model and not a defect of the model itself.

The sigmoidal $K_{\text{B}} \equiv 1/K_{\text{I}}$–pCa relationship is only one convenient way of describing thin filament regulation of myosin binding in solution. However, to implement thin filament regulation in models of muscle fiber contraction, it is desirable to develop a model in which these functions can be fitted by an allosteric reaction scheme which couples $K_{\text{I}}$ to the calcium binding kinetics to TnC (Geeves et al. 2011; Smith and Geeves 2003), which in the case of skeletal muscle requires binding of two calcium ions to the low-affinity sites. A similar approach can be applied to cardiac muscle, which requires binding of one calcium ion. This is especially important in studying the mechanics and physiology of muscle fibers driven by temporally variable Ca^2+^ concentration.

## Conclusions

We have shown that the CFC model can readily be extended to describe the regulation of myosin–actin kinetics by local configurations of the tropomyosin chain which are presumed to be in rapid equilibrium on the time scale of myosin binding. This has been demonstrated by fitting the time courses of experimental myosin binding data under a variety of conditions, including a range of calcium levels. A particularly convincing proof is obtained by plotting the detachment affinity of TnI used to fit the data against the experimental calcium concentration involved, giving practically identical curves for the excess-actin and excess-myosin data.

More generally, the Monte Carlo simulations presented here illustrate the three types of cooperativity present in the CFC model in ways not readily accessible by analytic methods. At high calcium, the dominant effect is myosin–myosin cooperation via shared kinks of the Tm–Tn chain. At low calcium, the dominant effect is inhibitory; myosin binding is inhibited by opposing kinks due to bound TnIs, with a large strain-energy cost for putting a bound myosin in a nearest-neighbor site. An additional inhibitory effect for myosin is the cooperative action of bound TnIs, which occurs because their ~38.5 nm (axial) spacing (where the Tm structural unit size is ~40 nm) is less than the size of a single kink, namely twice the chain persistence length of the confined chain, 1/*ξ*. However, TnI–TnI cooperativity does require kinks induced by TnI, and vanishes if *ϕ*_−_ is set to zero.

Further testing of the model is possible by using different isoforms of Tn and Tm. Changes in Tn (skeletal Tn for cardiac Tn) should alter the calcium dependence of S1 binding transients but are not predicted to alter the Tm flexibility. Different Tm isoforms have been reported to have different solution persistence lengths (Tm carrying point mutations associated with cardiomyopathies; D175N and E180G; Li et al. 2012; Loong et al. 2012; Sumida et al. 2008) and therefore may be expected to alter the flexibility of Tm on actin. These studies are underway.

## Electronic supplementary material

Below is the link to the electronic supplementary material.

Supplementary material 1 (DOC 7708 kb)

## Footnotes

1 A Markov process is a simple stochastic process in which the distribution of future states depends only on the present state and not on how it arrived in the present state.

2 Solution measurements of the initial rate of myosin binding to fully regulated actin as a function of calcium yield a Hill coefficient of 1.8; this value applies to the myosin-free filament and therefore argues against the existence of I–I interactions.
