# Supplementary material for: Cooperative regulation of myosin-S1 binding to actin filaments by a continuous flexible Tm–Tn chain
Source: Eur Biophys J. 2012 Oct 7;41(12):1015–32. doi: 10.1007/s00249-012-0859-8 (PMC3509328; doi:10.1007/s00249-012-0859-8)
Supplement: Supplementary file 1 — Supplementary material 1 (DOC 7708 kb) [file 249_2012_859_MOESM1_ESM.doc]

**Supplementary Material**

**Cooperative Regulation of Myosin-S1 Binding to Actin Filaments by a Continuous Flexible Tm-Tn Chain**

**Srboljub M. Mijailovich^†§^*, Oliver Kayser-Herold^†^, Xiaochuan Li^†^, Hugh Griffiths^‡^, and Michael A. Geeves^‡^**

**^†^**Dept. of Environmental Health, Harvard School of Public Health, Boston, MA 02115,

**^‡^**Dept. of Biosciences, University of Kent, Canterbury, Kent CT2 7NJ, U.K.,

**^§^**Tufts University, School of Medicine, Department of Medicine, *****Steward St. Elizabeth’s Medical Center, Boston, MA 02135

**Suppl. Fig. S1**

The best fits of our model to the excess actin and the excess S1 kinetic data compared to the best fits achieved by previous models to the same data set. We show fits using the Hill model as formulated by Chen et al. (2001), the Mckillop & Geeves model (1993) as formulated by (Mijailovich et al., 2012) and the current CFC model fits shown in Fig 4 of the main paper.

All three models describe the data well with little deviation between the experimental data and the fits. The Hill model, however, deviates at longer times in the case of excess S1 to actin concentration. This is entirely due to the lack of negative cooperativity on the Hill model. The negative cooperativity is required to fit an apparent slowing of the reaction as the actin filament becomes saturated with S1. We believe an introduction of this into the Hill model would result in equally good fits as achieved by current CFC model.

The ability to fit the data does not distinguish between the different models as pointed out in our previous papers (Geeves et al., 2011, Mijailovich et al., 2012). What does distinguish them is the ability to fit the whole data set with parameters that show simple calcium dependence. This is illustrated in Fig. S2 where we plot the values of K_B_ as a function of calcium concentration.

**Suppl. Fig. S2**

This shows the calcium dependence of K_B_ collected here compared to the values obtained in an earlier version of the CFC model (Geeves et al., 2011) and the McKillop and Geeves (McK-G) model as formulated by (Mijailovich et al., 2012). All three models predict the same mid point at pCa ~6.0 and a similar slope over the middle range (Hill coefficient). The McK-G model has higher values at pCa <5.5 but the system is not sensitive to values of Kb > 10, thus these differences are not significant. The Geeevs et al. (2011) data have lower values of K_B_ at low calcium due to the sensitivity of the system to the rate constant of TnI rebinding. At higher calcium concentrations the position of CFC is dominated by bound S1, so the rebinding rate constant of TnI become less important, thus the values of the equilibrium constant K_B_ become similar in both models.
